# Thermally controllable Mie resonances in a water-based metamaterial

**DOI:** 10.1038/s41598-019-41681-5

**Published:** 2019-04-01

**Authors:** Xiaqing Sun, Quanhong Fu, Yuancheng Fan, Hongjing Wu, Kepeng Qiu, Ruisheng Yang, Weiqi Cai, Nan Zhang, Fuli Zhang

**Affiliations:** 1 Research & Development Institute in Shenzhen, Key Laboratory of Space Applied Physics and Chemistry, Ministry of Education, and Department of Applied Physics, School of Natural and Applied Sciences, Northwestern Polytechnical University, Xi’an, 710072 China; 2 School of Mechanical Engineering, Northwestern Polytechnical University, Xi’an, 710072 China

## Abstract

Active control of metamaterial properties is of great significance for designing miniaturized and versatile devices in practical engineering applications. Taking advantage of the highly temperature-dependent permittivity of water, we demonstrate a water-based metamaterial comprising water cubes with thermally tunable Mie resonances. The dynamic tunability of the water-based metamaterial was investigated via numerical simulations and experiments. A water cube exhibits both magnetic and electric response in the frequency range of interest. The magnetic response is primarily magnetic dipole resonance, while the electric response is a superposition of electric dipole resonance and a smooth Fabry–Pérot background. Using temporal coupled-mode theory (TCMT), the role of direct scattering is evaluated and the Mie resonance modes are analyzed. As the temperature of water cube varies from 20 °C to 80 °C, the magnetic and electric resonance frequencies exhibit obvious blue shifts of 0.10 and 0.14 GHz, respectively.

## Introduction

Metamaterials have attracted considerable attention of researchers for the unique properties^1^. The electromagnetic properties of metamaterials are highly dependent on the geometry and arrangement of unit cells, providing additional freedom for designing the electromagnetic properties of materials^2^. Metamaterials have a wide range of applications, including perfect lenses^3,4^, invisible cloaks^5–7^, perfect absorbers^8,9^, polarization manipulators^10^, and nonlinear optical media^11,12^. Moreover, metamaterials are employed to imitate the characteristic features of quantum phenomena, e.g., Fano resonance^13^ and electromagnetically induced transparency^14^. At high frequencies, especially at optical frequencies, metallic resonators suffer from significant ohmic loss, impairing the performance of the metamaterial. As a result of low dielectric loss, dielectric resonators based on the principle of Mie resonance are a good alternative to metallic resonators^15–17^.

Active control of metamaterial properties is of great significance for designing miniaturized and versatile devices in practical engineering applications. According to the LC resonance model of metallic resonators, introducing active elements, e.g., varactor diodes, into metallic resonators is an effective method of tuning the resonance frequency of a metamaterial^18–22^. Influenced by the host material, the resonance of metamaterials can be tuned by integrating tunable materials, e.g., liquid crystals^23,24^, phase change materials^25,26^, semiconductor materials^27^, and graphene^28–30^, into unit cells. Furthermore, using resonators made of tunable materials is another means of developing tunable metamaterials^31,32^.

Water exhibits high permittivity in the microwave band and is therefore a good candidate for dielectric metamaterials. To date, most studies on water-based metamaterials have focused on broadband absorbers owing to the large dielectric loss of water^33–37^. However, after inspecting the dielectric dispersion of water, it was found that the dielectric loss decreases with decreasing frequency^38^. Consequently, low dielectric loss can be obtained at low frequencies, enabling water-based metamaterials with Mie resonance of high quality factor. Andryieuski *et al*. theoretically demonstrated thermal, mechanical and gravitational tunability of magnetic and electric resonances in a metamaterial consisting of periodically positioned water-filled reservoirs, opening the door of water-based metamaterials^39^. Herein, in combination with the highly temperature-dependent permittivity of water, we present a water-based metamaterial and investigate the thermally tunable Mie resonance via simulations, experiments, and temporal coupled-mode theory (TCMT). With the standard S-parameter retrieval method, the effective permeability and permittivity are retrieved to describe the macroscopic properties of a water cube array; through full-wave simulation and numerical calculation, the microscopic response of a water cube is quantitatively analyzed in detail. Using TCMT, the role of direct scattering in the formation of the spectral line shape of a water cube is elucidated, and the electric and magnetic resonance modes are analyzed. Finally, the Mie resonance of a water cube at a temperature ranging from 20 °C to 80 °C is investigated to confirm the thermal tunability of a water-based metamaterial.

## Results

### Design and modeling

**Figure 1:**
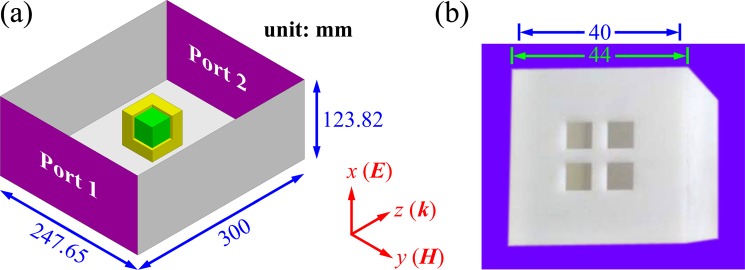
Schematic (**a**) and photograph (**b**) of a water cube placed at the center of a standard rectangular waveguide of BJ9 with cross-section of 247.65 mm × 123.82 mm. The shape of the water cube is maintained with a 3D-printed resin shell with thickness of 2 mm and permittivity of 3.5 × (1 − 0.1j)^47^. Four square holes are drilled in the resin shell for injecting water. To display the resin shell and water cube, one corner of the resin shell is removed in the schematic. The yellow shell and green cube represent the resin shell and water cube, respectively.

According to the Debye model of the frequency-dependent permittivity of water in microwave band^38^, the dielectric loss of water decreases with decreasing frequency. Therefore, low loss can be attained at low frequencies. Moreover, the permittivity of water is highly sensitive to temperature, providing a basis for developing water-based metamaterials with a thermally tunable electromagnetic response. In our designs, a water cube is used as a building block of water-based metamaterials, as shown in Fig. 1. The electromagnetic response of the water cube in the frequency range 0.70–1.15 GHz is investigated in a standard rectangular waveguide of BJ9, and the scattering parameters are simulated with the commercial software CST Microwave Studio and measured with a vector network analyzer (AV3629D). In the model illustrated in Fig. 1a, the PEC boundaries are applied to simulate the electromagnetic response of a water cube in a standard rectangular waveguide. In experiments, the incident power of wave port is set to −25 dBm so that the nonlinear response of water cube arising from absorption is negligible.

### Mie resonance of a water cube

**Figure 2:**
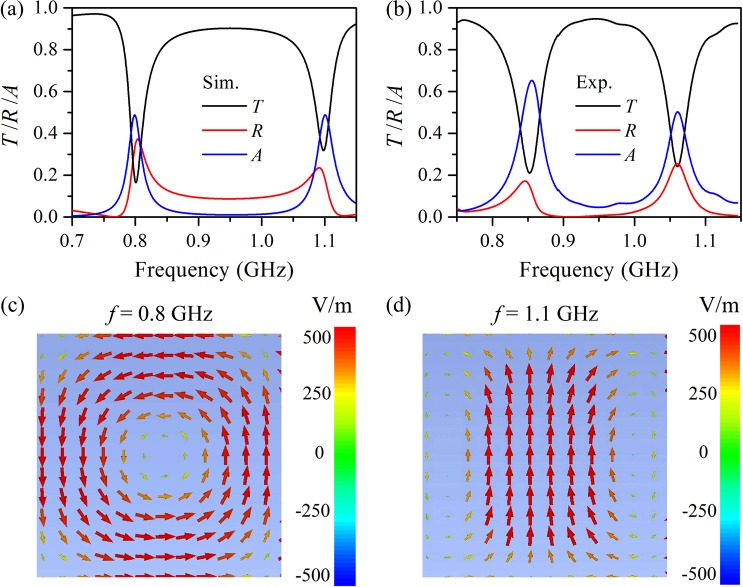
Transmission, reflection, and absorption spectra of a water cube with edge length of 40 mm at 80 °C acquired by both simulations (**a**) and experiments (**b**). Electric field distribution at the frequency of magnetic resonance (**c**) and electric resonance (**d**) on the cross section parallel to *xz* plane through the center of the water cube.

Figure 2a shows the simulated transmission, reflection, and absorption spectra of a water cube with edge length of 40 mm at 80 °C. It is clear that two dips appear with transmittance of 0.17 and 0.32 at 0.80 and 1.1 GHz, respectively, in the transmission spectrum, indicating the Mie resonance of the water cube. According to Mie scattering theory, the resonance at 0.80 GHz is a magnetic response and the resonance at 1.1 GHz is an electric response since the magnetic resonance frequency is lower than the electric one in the first-order scattering of a water cube. Moreover, both the reflection and absorption spectra of the water cube manifest a peak near each Mie resonance; the absorbance is negligible at frequencies far from the Mie resonance, but this is not the case for the reflectance. It should be noted that the reflection spectrum exhibits non-Lorentzian line shape, the reason for which will be detailedly explained later. Figure 2b shows the experimental results of the transmission, reflection, and absorption spectra, and they match well with the numerical results presented in Fig. 2a; the discrepancy between them mainly arises from the imperfect sample fabrication in experiment and the inaccurate permittivity of water in simulation.

To further interpret the Mie resonance of the water cube, the local electric field at the Mie resonance frequency is calculated and plotted in Fig. 2c,d. The displacement current is proportional to the electric field and thus Fig. 2c,d also represent the displacement current. Figure 2c displays the circulating displacement current, which leads to a magnetic dipole moment and thus a magnetic dipole response, at 0.80 GHz; the linear displacement current at 1.1 GHz shown in Fig. 2d results in an electric dipole moment and therefore an electric dipole response.

**Figure 3:**
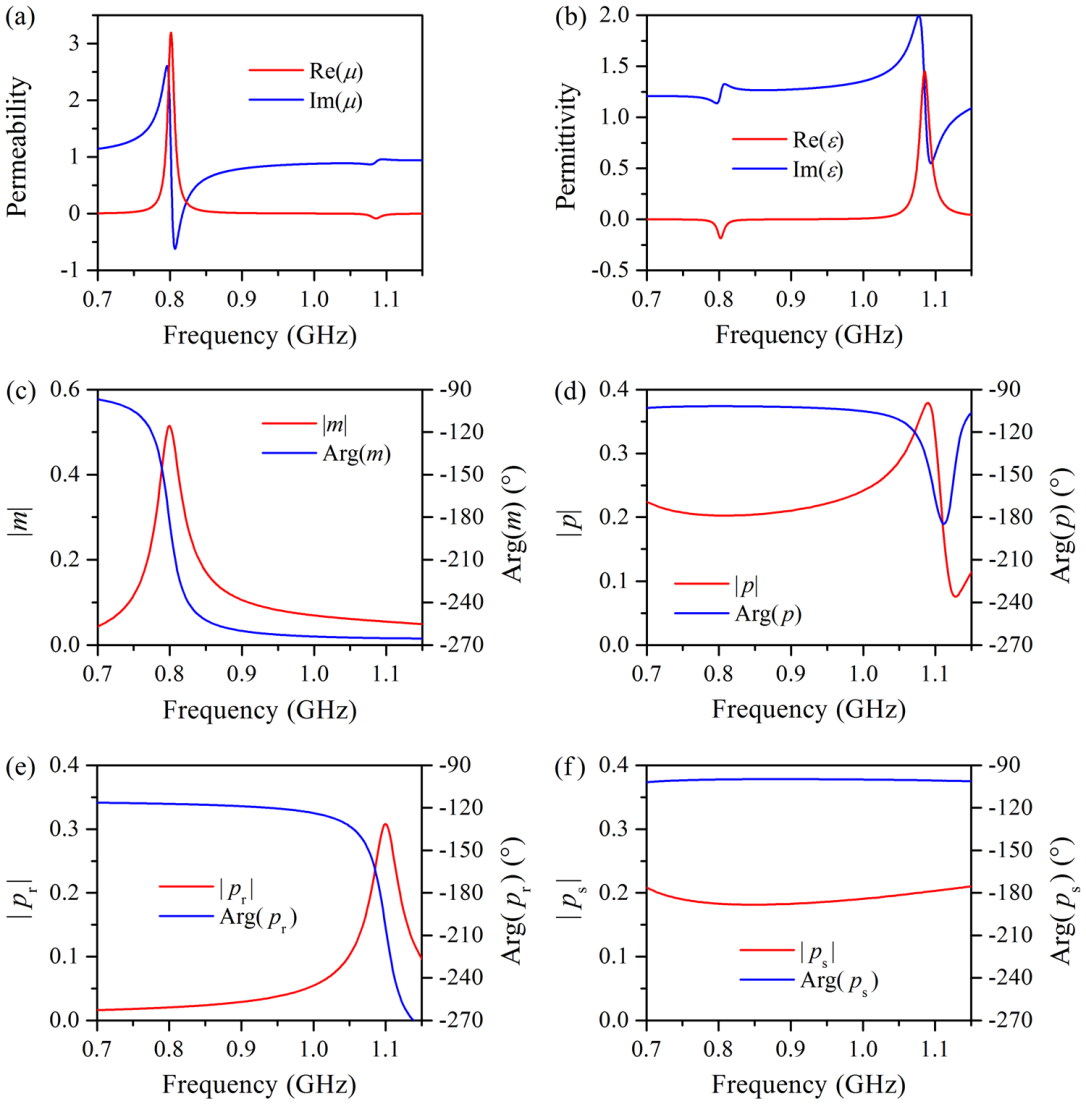
Retrieved effective permeability (**a**) and permittivity (**b**) of a water cube array with the standard *S*-parameter retrieval method. Calculated magnetic dipole moment (**c**) and electric dipole moment (**d**), which are normalized to the effective magnetic and electric dipole sources, respectively, of a water cube inside a standard rectangular waveguide of BJ9. Electric dipole resonance (**e**) and smooth Fabry–Pérot background (**f**) involved in the electric dipole response of the water cube.

To describe the macroscopic electromagnetic properties of water-based metamaterials, the effective permeability and permittivity of the water cube array are retrieved with the standard *S*-parameter retrieval method, as shown in Fig. 3a,b. It is evident that the permeability and permittivity spectra display resonances around 0.80 and 1.1 GHz, respectively, which are in accordance with the frequency of transmission dips in Fig. 2a; the minimum of permeability and permittivity reach −0.62 and 0.55, respectively. It is noteworthy that the permittivity at frequencies far from electric resonance is appreciably more than unity, indicating that the electric response of the water cube is actually a superposition of the electric dipole resonance and a smooth Fabry–Pérot background^40^. In contrast, the permeability spectrum reveals that the magnetic response of the water cube is primarily the magnetic dipole resonance. By tuning the geometric parameters of the water cube array, desired permeability and permittivity can be realized. Furthermore, to study the microscopic response of the water cube, the magnetic and electric dipole moments of the water cube placed inside a standard rectangular waveguide of BJ9 are calculated and normalized to the effective magnetic and electric dipole sources, respectively, for the sake of obvious physical meaning. Figure 3c,d show the normalized magnetic dipole moment *m* and electric dipole moment *p* of a water cube. It is evident that the *m*-spectrum exhibits a resonance near 0.80 GHz and that the magnitude of *m* reaches a maximum of 0.51. The phase of *m* is approximately −180° at the magnetic resonance frequency, indicating that the scattering wave of the magnetic dipole destructively interferes with the incident wave and leads to the transmission dip at 0.80 GHz. Nevertheless, the *p*-spectrum is strikingly different from the *m*-spectrum in terms of both the magnitude and phase. The magnitude of *p* approaches a maximum of 0.38 and a minimum of 0.076 at 1.09 and 1.13 GHz, respectively. Moreover, the phase of *p* does not monotonically decrease but exhibits a minimum at 1.11 GHz as the frequency increases. As mentioned above, the *p*-spectrum of the water cube is the electric dipole resonance superimposed on a smooth Fabry–Pérot background. To extract the electric dipole resonance from the *p*-spectrum, we analyze the *p*-spectrum in a narrow frequency range around the electric resonance frequency, for example, 1.05–1.15 GHz, where the smooth Fabry–Pérot background is approximately a linear function of the frequency. Now, the *p*-spectrum can be expressed as

1 $$\begin{array}{rcl}p(f) & = & {p}_{r}(f)+{p}_{s}(f)\\  & = & -j{a}_{1}/({{f}_{0}}^{2}-{f}^{2}+j\,2\beta f)+{a}_{2}f+{a}_{3}\end{array}$$

where *a*_1_, *a*_2_, *a*_3_, *ω*_0_, and *β* are parameters to be determined. Through least squares fitting, the electric dipole resonance *p*_r_(*f*) can be determined, as shown in Fig. 3e, and is valid in the entire frequency range of interest. It is obvious that the electric dipole resonance occurs at 1.09 GHz, but the corresponding phase is significantly less than −180° because the smooth Fabry–Pérot background contributes to the local field, and hence, parameter *a*_1_ is complex, i.e. 0.0106 − 0.0048j GHz^2^. The smooth Fabry–Pérot background *p*_s_(*f*) in the frequency range of interest is the difference between the *p*-spectrum and the electric dipole resonance, as shown in Fig. 3f, and resembles the response of a dielectric slab. It is *p*_s_(*f*) that results in the non-Lorentzian spectral line shape of the water cube.

### TCMT-based analysis of Mie resonance

The origin of the non-Lorentzian spectral line shape of a water cube can also be explained by TCMT. Each water cube can be considered as a resonator system comprising electric and magnetic dipole resonators. TCMT is then employed to understand the origin of spectral features and acquire the parameters of the resonator system, though mode coupling does not exist in a water cube. The dynamic equation of the resonance amplitude can be written as^41–46^

2 $$\frac{{\rm{d}}}{{\rm{d}}t}(\begin{array}{c}{a}_{m}\\ {a}_{e}\end{array})=(j{\rm{\Omega }}-{{\rm{\Gamma }}}_{{\rm{e}}}-{{\rm{\Gamma }}}_{{\rm{i}}})(\begin{array}{c}{a}_{m}\\ {a}_{e}\end{array})+{D}^{{\rm{T}}}(\begin{array}{c}{s}_{1+}\\ {s}_{2+}\end{array})$$

3 $$(\begin{array}{c}{s}_{1-}\\ {s}_{2-}\end{array})=C(\begin{array}{c}{s}_{1+}\\ {s}_{2+}\end{array})+D(\begin{array}{c}{a}_{m}\\ {a}_{e}\end{array})$$

where $${\rm{\Omega }}=(\begin{array}{cc}{\omega }_{1} & 0\\ 0 & {\omega }_{2}\end{array})$$, $${{\rm{\Gamma }}}_{{\rm{e}}}=(\begin{array}{cc}{{\rm{\Gamma }}}_{{\rm{e}}1} & 0\\ 0 & {{\rm{\Gamma }}}_{{\rm{e}}2}\end{array})$$, $${{\rm{\Gamma }}}_{{\rm{i}}}=(\begin{array}{cc}{{\rm{\Gamma }}}_{{\rm{i1}}} & 0\\ 0 & {{\rm{\Gamma }}}_{{\rm{i}}2}\end{array})$$, $$C=(\begin{array}{cc}{C}_{11} & {C}_{21}\\ {C}_{21} & {C}_{11}\end{array})$$, and $$D=(\begin{array}{cc}\sqrt{{{\rm{\Gamma }}}_{{\rm{e}}1}}{e}^{j{\varphi }_{1}} & \sqrt{{{\rm{\Gamma }}}_{{\rm{e}}2}}{e}^{j{\varphi }_{2}}\\ -\sqrt{{{\rm{\Gamma }}}_{{\rm{e}}1}}{e}^{j{\varphi }_{1}} & \sqrt{{{\rm{\Gamma }}}_{{\rm{e}}2}}{e}^{j{\varphi }_{2}}\end{array})$$

The meaning of the denotations in above formulas is as follows: *a*_m_ and *a*_e_ are the normalized amplitudes of the magnetic and electric dipole resonators, respectively, such that |*a*_m_|^2^ and |*a*_e_|^2^ correspond to the energy inside the resonators; *s*_1+_ and *s*_2+_ (or *s*_1−_ and *s*_2−_) are the normalized amplitudes of the incoming (or outcoming) waves at port 1 and port 2, respectively, such that |*s*_1+_|^2^ and |*s*_2+_|^2^ (or |*s*_1−_|^2^ and |*s*_2−_|^2^) correspond to the power carried by the incoming (or outcoming) waves; because the magnetic and electric dipole modes do not couple with each other, both the matrices Ω and Г_e_ are diagonal; *ω*_1_, Г_e1_ and Г_i1_ (or *ω*_2_, Г_e2_ and Г_i2_) are the resonance frequency, radiative decay, and nonradiative decay rates of the magnetic (or electric) dipole resonator, respectively; *C* is the direct scattering matrix in the absence of resonators; *D* represents the coupling between resonance modes and incoming/outcoming waves; *D*_11_ (or *D*_21_) represents the coupling between the magnetic dipole resonator and the incoming/outcoming waves at port 1 (or port 2); similarly, *D*_12_ (or *D*_22_) represents the coupling between the electric dipole resonator and the incoming/outcoming waves at port 1 (or port 2). The resonator system has a mirror symmetry: the magnetic and electric dipole modes are of odd and even symmetry, respectively; thus, *D*_11_ = −*D*_21_ and *D*_12_ = *D*_22_.

The matrices *D* and Г_e_ are not independent but constrained by the relation *D*^+^*D* = 2Г_e_. Therefore, $|{D}_{11}|=\sqrt{{{\rm{\Gamma }}}_{{\rm{e}}1}}$ and $|{D}_{22}|=\sqrt{{{\rm{\Gamma }}}_{{\rm{e}}2}}$ so that ${D}_{11}=\sqrt{{{\rm{\Gamma }}}_{{\rm{e}}1}}{e}^{j{\varphi }_{1}}$ and ${D}_{22}=\sqrt{{{\rm{\Gamma }}}_{{\rm{e}}2}}{e}^{j{\varphi }_{2}}$. Strictly, the direct scattering matrix *C* is frequency dependent and thus cannot be determined by the fitting method. In fact, *C* lies with the smooth Fabry–Pérot background *p*_s_(*f*) in the electric dipole response of the water cube and is expressed as *C*_11_ = *p*_s_(*f*) and *C*_21_ = 1 + *p*_s_(*f*) because the reflected wave is the scattering wave and the transmitted wave is a superposition of the incident and scattering waves.

When the resonator system is excited by a monochromatic wave, the scattering matrix of the water cube can be expressed as

4 $$S=C+D{[{\rm{j}}\omega I-(j{\rm{\Omega }}-{{\rm{\Gamma }}}_{{\rm{i}}}-{{\rm{\Gamma }}}_{{\rm{e}}})]}^{-1}{D}^{{\rm{T}}}$$

Hence, the reflection and transmission spectra are expressed as follows:

5 $$\{\begin{array}{c}{S}_{11}={C}_{11}+\frac{{{\rm{\Gamma }}}_{{\rm{e}}1}{e}^{j2{\varphi }_{1}}}{{\rm{j}}\omega -{\rm{j}}{\omega }_{1}+{{\rm{\Gamma }}}_{{\rm{i}}1}+{{\rm{\Gamma }}}_{{\rm{e}}1}}+\frac{{{\rm{\Gamma }}}_{{\rm{e}}2}{e}^{j2{\varphi }_{2}}}{{\rm{j}}\omega -{\rm{j}}{\omega }_{2}+{{\rm{\Gamma }}}_{{\rm{i}}2}+{{\rm{\Gamma }}}_{{\rm{e}}2}}\\ {S}_{21}={C}_{21}-\frac{{{\rm{\Gamma }}}_{{\rm{e}}1}{e}^{j2{\varphi }_{1}}}{{\rm{j}}\omega -{\rm{j}}{\omega }_{1}+{{\rm{\Gamma }}}_{{\rm{i}}1}+{{\rm{\Gamma }}}_{{\rm{e}}1}}+\frac{{{\rm{\Gamma }}}_{{\rm{e}}2}{e}^{j2{\varphi }_{2}}}{{\rm{j}}\omega -{\rm{j}}{\omega }_{2}+{{\rm{\Gamma }}}_{{\rm{i}}2}+{{\rm{\Gamma }}}_{{\rm{e}}2}}\end{array}$$

It is seen that both *S*_11_ and *S*_21_ contain three terms: the first term represents direct scattering; the latter two terms represent magnetic and electric dipole scattering, which are standard Lorentzian resonances. The direct scattering terms *C*_11_ and *C*_21_ result in the deviations of *S*_11_ and *S*_21_, respectively, from the Lorentzian line shape.

**Figure 4:**
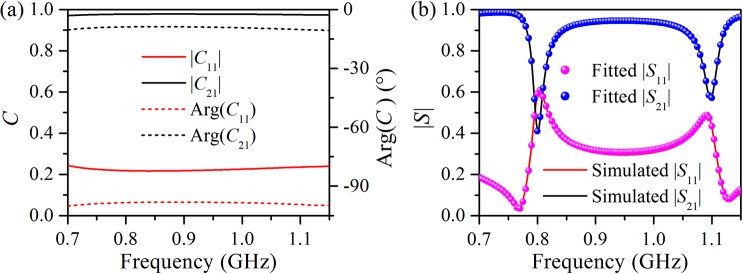
(**a**) Direct scattering parameters *C*_11_ and *C*_21_ obtained via *p*_s_(*f*). (**b**) Reflection and transmission spectra of a water cube, as predicted by the temporal coupled-mode theory. For comparison, the simulated result is plotted in (**b**).

The direct scattering parameters *C*_11_ and *C*_21_ are calculated from the smooth Fabry–Pérot background *p*_s_(*f*), as shown in Fig. 4a. It is apparent that *C*_11_ and *C*_21_ are slowly varying functions of frequency, and exhibit high transmission and low reflection. The direct reflectance *C*_11_ and transmittance *C*_21_ have an average of $0.227\angle -99^\circ$ and $0.976\angle -9.5^\circ$, respectively. Although the water cube has nonnegligible loss, careful examination reveals that the direct scattering matrix *C* is approximately a unitary matrix, i.e. *C*^+^*C* ≈ *I*, and therefore, the direct scattering is nearly lossless. Thus, the constraint relation CD^*^ = −*D* approximately holds and leads to relations *C*_11_–*C*_21_ = −${e}^{j2{\varphi }_{1}}$ and *C*_11_–*C*_21_ = −${e}^{j2{\varphi }_{2}}$. Considering the slow variation of *C*_11_ and *C*_21_ with frequency, the average of *C*_11_ and *C*_21_ can be utilized to calculate *ϕ*_1_ and *ϕ*_2_ as *ϕ*_1_ = −178° and *ϕ*_1_ = −101°.

After calculating the direct scattering matrix *C*, the parameters of the resonator system can be acquired by fitting the expression of *S*_11_ and *S*_21_ in formula (5) to the simulated reflection and transmission spectra of the water cube shown in Fig. 2a. The fitting can be carried out with the command *lsqcurvefit* in MATLAB and the fitted parameters are the following:

*ω*_1_ = 2π × 0.7983 × 10^9^ rad/s, *ω*_2_ = 2π × 1.1008 × 10^9^ rad/s,

Г_e1_ = 2π × 0.0074 × 10^9^ rad/s, Г_e2_ = 2π × 0.0067 × 10^9^ rad/s,

Г_i1_ = 2π × 0.0055 × 10^9^ rad/s, Г_i2_ = 2π × 0.0095 × 10^9^ rad/s,

*ϕ*_1_ = −176°, and *ϕ*_2_ = −102°.

It is evident that the fitting resonance frequencies *ω*_1_ and *ω*_2_ are highly consistent with the two transmission dips (or reflection peaks) of the water cube shown in Fig. 3a. The radiative and nonradiative decay rates of each resonance mode are comparable, and they are both much less than the corresponding resonance frequency. Moreover, the values of *ϕ*_1_ and *ϕ*_2_ obtained by the two methods match well; the phase difference between the scattering wave and incident wave is −176° × 2 + 180° = −172° and −102° × 2 + 180° = −204° for the magnetic and electric dipole resonances, respectively. The magenta and blue solid circles in Fig. 4b show the reflection and transmission spectra of the water cube, as predicted by TCMT. It is clear that TCMT exactly reproduces the simulated reflection and transmission spectra of the water cube well in the entire frequency range of interest, indicating that the interaction between the water cube and the incident wave is well described by TCMT.

**Figure 5:**
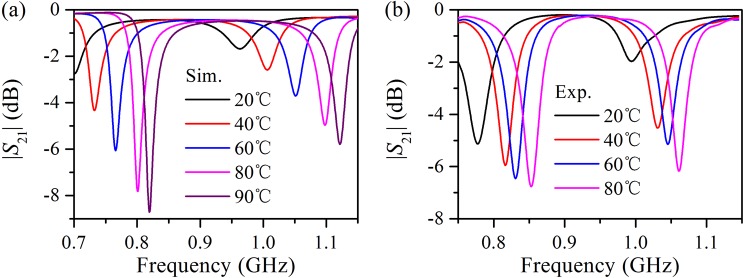
Transmission spectra of the water cube with edge length of 40 mm acquired via both simulations (**a**) and experiments (**b**) at various temperatures.

To examine the thermal tunability of the water-based metamaterial, the transmission spectra of a water cube from 20 °C to 80 °C are numerically simulated and experimentally measured, as shown in Fig. 5a,b. The simulated results illustrate that when the temperature of the water cube increases from 20 °C to 80 °C, the magnetic resonance frequency accordingly shifts from 0.70 to 0.80 GHz, a blue shift of 0.10 GHz, with the corresponding transmittance decreasing from −2.7 to −7.8 dB. Meanwhile, the electric resonance frequency shifts from 0.96 to 1.1 GHz, a blue shift of 0.14 GHz, with the corresponding transmittance decreasing from −1.7 to −5.0 dB. The measured results agree well with the simulated results, confirming the effective control and tunability of the electromagnetic properties of water-based metamaterials with temperature. As stated earlier, the thermal tunability of the water-based metamaterial originates from the temperature-dependent permittivity and dielectric loss of water. As the temperature rises, the permittivity of water decreases, and hence, the resonance frequency of the water cube increases. Meanwhile, the dielectric loss of water decreases, and thus, the transmission dip near the resonance frequency becomes deeper, indicating a higher quality factor of the Mie resonance.

**Figure 6:**
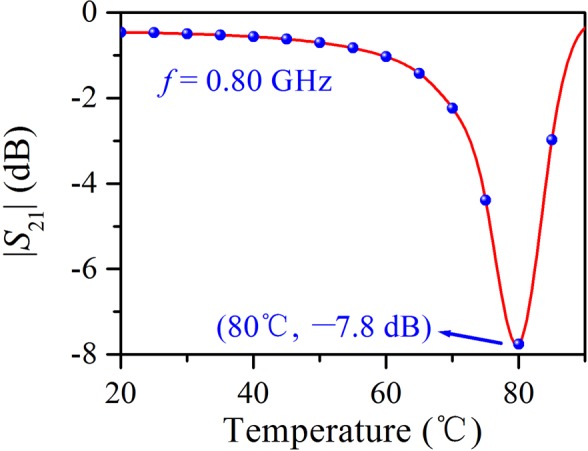
Relation between the transmittance of the water cube with edge length of 40 mm and the temperature for a 0.80 GHz microwave acquired via simulations.

Because of the sharp transmission dip arising from the electric and magnetic resonances, the transmittance of the water cube is subject to considerable variation around the resonance frequency when the resonance frequency shifts with temperature. At the frequency of 0.80 GHz, for example, the transmittance of the water cube first decreases from −0.46 dB to a minimum of −7.8 dB and then increases to −0.97 dB as the temperature varies from 10 °C to 90 °C, as shown in Fig. 6, demonstrating a modulation depth of 94%. From this point of view, the water cube can act as a microwave modulator. It is worth noting that the transmittance of the water cube is only sensitive to temperature when the microwave frequency corresponds to the center temperature of the effective temperature range for controlling the transmittance of the water cube. For example, when the temperature is 80 °C, the magnetic resonance of the water cube occurs around 0.80 GHz. Thus, when around 80 °C, the temperature is capable of effectively controlling the transmittance of the water cube for a 0.80 GHz microwave.

## Conclusions

In summary, we presented a water-based metamaterial with thermal tunability and investigated the dynamically tunable Mie resonances via simulations, experiments, and TCMT. The water cube exhibited both the magnetic and electric resonances in the frequency range of interest. With the standard *S*-parameter retrieval method, the effective permeability and permittivity were retrieved to describe the macroscopic properties of the water cube array; through full-wave simulation and numerical calculation, the microscopic response of the water cube was also quantitatively analyzed in detail. The magnetic response of the water cube is primarily the magnetic dipole resonance; however, the electric response is a superposition of the electric dipole resonance and a smooth Fabry–Pérot background. Using TCMT, the role of direct scattering in the formation of the spectral line shape of the water cube was elaborated and the electric and magnetic resonance modes were analyzed; moreover, the parameters of the resonator system were retrieved, and the simulated reflection and transmission spectra were exactly reproduced. As the temperature of the water cube varies from 20 °C to 80 °C, the magnetic and electric resonance frequencies exhibited obvious blue shifts of 0.10 and 0.14 GHz, respectively, owing to the decreasing permittivity of water, confirming the dynamically tunable Mie resonance of the water cube; around the resonance frequency of the water cube, the transmittance experiences a conspicuous modulation and the modulation depth is up to 94%. The results of this work may be useful for applications, including temperature sensing and microwave modulation.

## Methods

In experiments, ultrapure water was used; after boiled, the ultrapure water was injected into the resin shell; the water cube was fixed at the center of rectangular waveguide by a foam fixture. The temperature of ultrapure water was measured with a thermocouple, and the *S* parameters of water cube were measured during cooling with a vector network analyzer (AV3629D). The full-wave numerical simulation software CST microwave studio was employed to analyze the spectral response and field distribution. Open and PEC boundary conditions were set perpendicular and parallel to the wave propagating direction (*z*-direction), respectively.

## Supplementary information

Thermally controllable Mie resonances in a water-based metamaterial

## Data Availability

The data supporting the findings of this study are available within the article and its Supplementary Information files. All other relevant source data are available from the corresponding author upon reasonable request.
