# Supplementary material for: Thermally controllable Mie resonances in a water-based metamaterial
Source: Sci Rep. 2019 Apr 1;9:5417. doi: 10.1038/s41598-019-41681-5 (PMC6443793; doi:10.1038/s41598-019-41681-5)
Supplement: Supplementary file 1 — Thermally controllable Mie resonances in a water-based metamaterial [file 41598_2019_41681_MOESM1_ESM.pdf]

Thermally controllable Mie resonances in a water-based metamaterial

Xiaqing Sun,[1] Quanhong Fu,[1] Yuancheng Fan,[1] Hongjing Wu,[1] Kepeng Qiu,[2] Ruisheng Yang,[1] Weiqi Cai,[1] & Fuli Zhang[1]

[1]Research & Development Institute in Shenzhen, Key Laboratory of Space Applied Physics and Chemistry, Ministry of Education, and Department of Applied Physics, School of Natural and Applied Sciences, Northwestern Polytechnical University, Xi'an 710072, China.

[2]School of Mechanical Engineering, Northwestern Polytechnical University, Xi'an 710072, China.

email: fuli.zhang@nwpu.edu.cn

Contents

1. Temperature-dependent dielectric dispersion of water
2. Effect of temperature on the reflection and absorption of water cube
3. Dependence of the transmission on the size of water cube
4. Influence of temperature on the magnetic and electric dipole moments of water cube

Temperature-dependent dielectric dispersion of water

The frequency-dependent permittivity of water in microwave band is described by the Debye model [1]

$$\varepsilon(\omega, T_{\text{water}}) = \varepsilon_\infty(T_{\text{water}}) + \frac{\varepsilon_0(T_{\text{water}}) - \varepsilon_\infty(T_{\text{water}})}{1 + j\omega\tau(T_{\text{water}})}$$

where ε_∞, ε_0, and τ are the high-frequency permittivity, static permittivity, and relaxation time, respectively, and they all depend on the temperature of water. The permittivity spectra of water at different temperature are shown in Fig. S1. It is demonstrated that the dielectric loss of water reduces with the decrement of frequency, and therefore low loss can be attained at low frequencies; moreover, the permittivity of water is highly sensitive to the temperature, providing a basis for developing water-based metamaterials with thermally tunable electromagnetic response. For the sake of quality factor of Mie resonance, we investigate the electromagnetic response of water-based metamaterials inside the standard rectangular waveguide of BJ9 because water is of low loss at the operating frequency of BJ9.

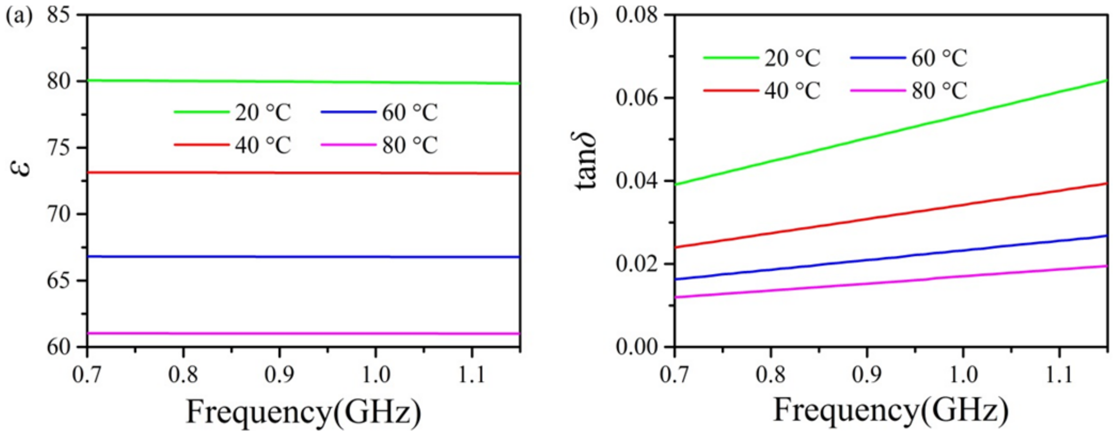

Figure S1. Real part (a) and loss tangent (b) of permittivity of water in the frequency range of 0.70~1.15 GHz at the temperature from 20°C to 80°C.

Effect of temperature on the reflection and absorption of water cube

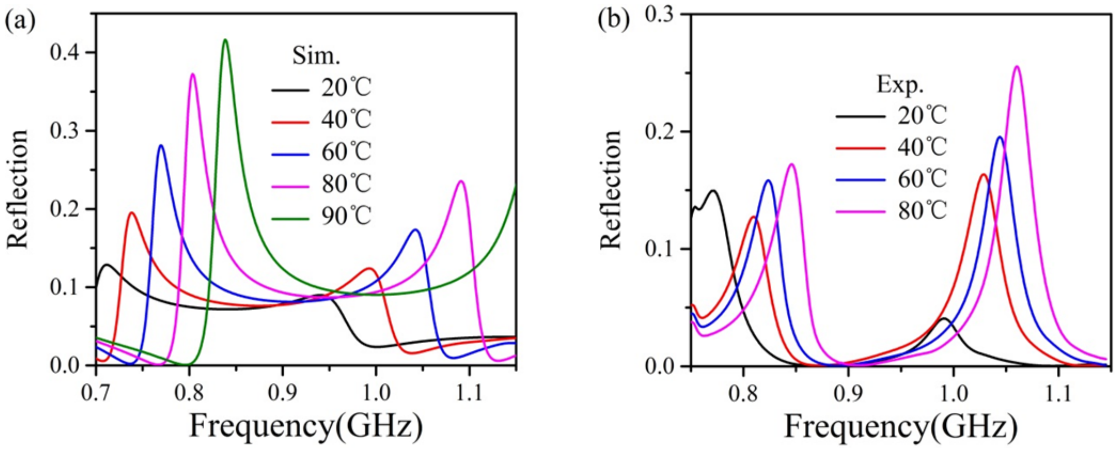

Figure S2. Reflection spectra of the water cube with edge length of 40 mm acquired via simulations (a) and experiments (b) at various temperatures.

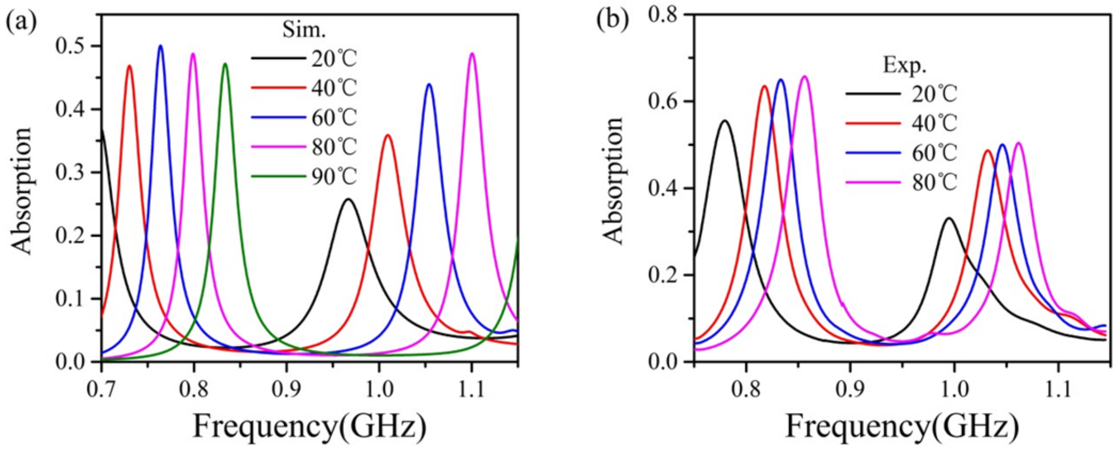

Figure S3. Absorption spectra of the water cube with edge length of 40 mm acquired via simulations (a) and experiments (b) at various temperatures.

Dependence of the transmission on the size of water cube

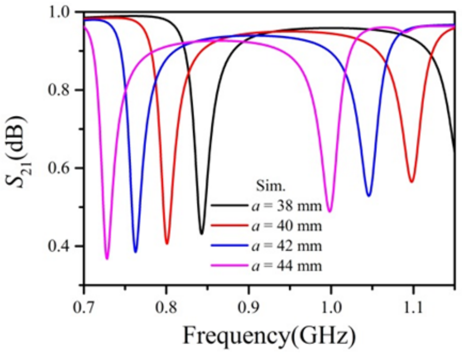

Figure S4. Transmission spectra of the water cubes with edge length *a* from 38 mm to 44 mm at the temperature of 80°C.

Influence of temperature on the magnetic and electric dipole moments of water cube

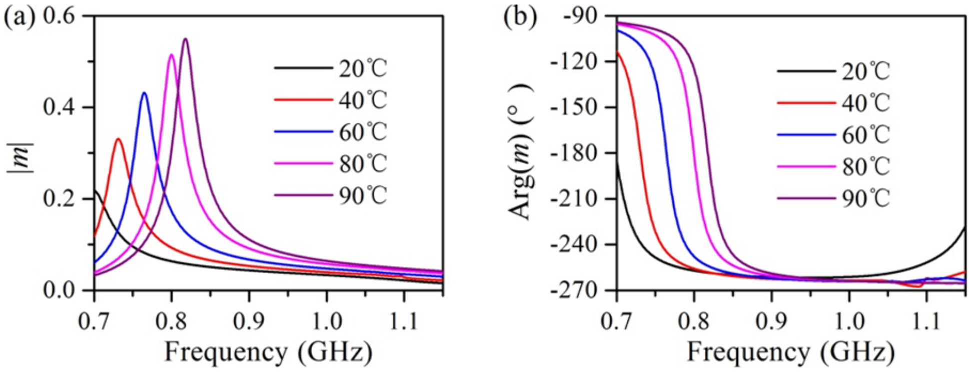

Figure S5. Magnitude (a) and phase (b) of the magnetic dipole moment of the water cube with edge length of 40 mm at various temperatures.

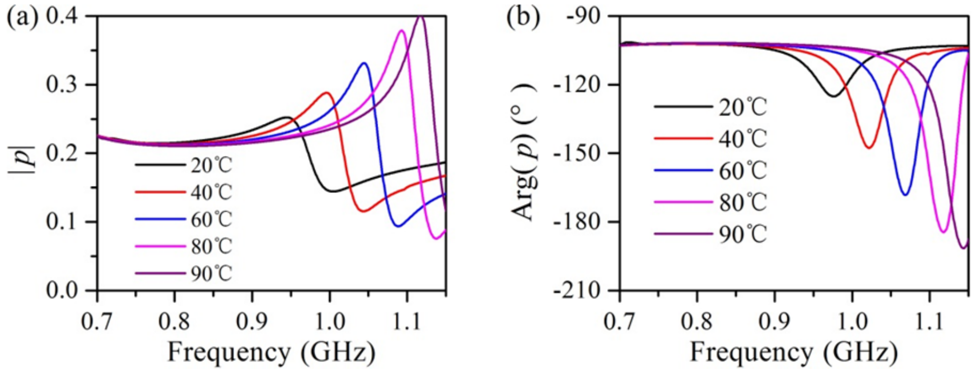

Figure S6. Magnitude (a) and phase (b) of the electric dipole moment of the water cube with edge length of 40 mm at various temperatures.

References

[1] Ellison, W. J. Permittivity of pure water, at standard atmospheric pressure, over the frequency range 0-25 THz and the temperature range 0-100°C. *J. Phys. Chem. Ref. Data* **36**, 1-18 (2007).
